# Maturity, Safety, and Equity of AI-Enabled Systems and Triage in Integrated Primary Care

**DOI:** 10.2196/97341

**Published:** 2026-05-14

**Authors:** Siaw-Teng Liaw

**Affiliations:** 1 Discipline of General Practice, School of Clinical Medicine and School of Population Health, UNSW Sydney, 24 Kelvinside St, Balwyn North, Victoria, 3104, Australia, 61 407818281

**Keywords:** artificial intelligence, AI, triage, primary care, general practice, patient safety, health equity, real-world evidence, clinical decision support, digital maturity, Quintuple Aim

## Abstract

Artificial intelligence (AI)–enabled systems must simultaneously improve the Quintuple Aim and digital health maturity, including equitable access to and quality and interoperability of data, tools, agents, and services. This requires a comprehensive sociotechnical and global approach to cocreation, management, and governance for individuals and organizations in the ecosystem.

Alamoudi et al [1] described the potential of artificial intelligence (AI)–enabled triage, lamented the lack of credible evidence, criticized the lack of real-world and equity-stratified outcomes, and proposed a sociotechnical approach to a “practical agenda for evaluating and governing AI-enabled triage in primary care that integrates real-world safety outcomes, equity-stratified performance reporting, and sociotechnical implementation and surveillance.” While commendable, equity aims must be set within the Quintuple Aim, which includes the cost-effective care of the individual and population, patient satisfaction, provider well-being, and equitable health for all [2], and the AI ethics and governance principles proposed by the World Health Organization (WHO) [3].

AI-enabled triage systems collect and analyze patient data to determine the urgency of care required, provide a risk assessment, and recommend a triage level, including self-care, routine review, urgent care, or emergency referral. This definition means that the management, governance, and evaluation of AI-enabled triage systems in primary care require an integrated approach where data, tools, agents, services, and systems are integrated and interoperable with those in other parts of the health ecosystem [4].

The learning and reasoning models underlying AI-enabled triage in general/family practice and primary care must reflect their patient-centered biopsychosocial diagnostic process, including making a probability diagnosis, excluding dangerous things, identifying differentials, and elucidating “hidden” patient agendas. This requires good quality models, algorithms, and data that are consistent with local community and institutional values, benchmarks, and priorities.

Therefore, this sociotechnical agenda must be implemented within a conceptual framework that includes improving the maturity, quality, and interoperability of the essential digital health foundations to enable the individual, organization, community, and ecosystem to adopt and integrate AI-enabled systems to achieve the Quintuple Aim successfully and sustainably.

However, the rapid deployment of AI-enabled systems despite a low evidence base for safety, bias, and quality [1,5], and the low digital health maturity (DHM) of individuals, organizations, and ecosystems raises concerns about inadequate digital transformation for health services adopting AI-enabled systems to achieve the Quintuple Aim [6].

The Digital Health Profile and Maturity Assessment Toolkit (DHPMAT) was developed to create a digital health profile and assess the DHM of health organizations and ecosystems. The digital health profile and DHM are then aligned with national, subnational, or organizational priorities to develop a realistic road map for adopting, managing, governing, evaluating, improving, and sustaining digital health interventions [4,7].

Figure 1 illustrates the use of DHPMAT to assess the sociotechnical and financial readiness of citizens, digital health professionals, and organizations to adopt AI-enabled systems, including triage, in the integrated health ecosystem. Readiness to implement digital health systems is determined by assessing the maturity of five essential digital health foundations (Figure 1, rows): information and communication technology, Internet-of-Things, or AI infrastructure; digital public goods; platform for information sharing; enabling environment; and quality improvement, measurement, monitoring, and evaluation. The five linear stages of DHM (Figure 1, columns) describe an initial adapting and assessing stage, which matures to controlling, standardizing, optimizing, and innovating stages. Standardizing at the center of Figure 1 (bolded in red) is the keystone stage [4].

**Figure 1.**
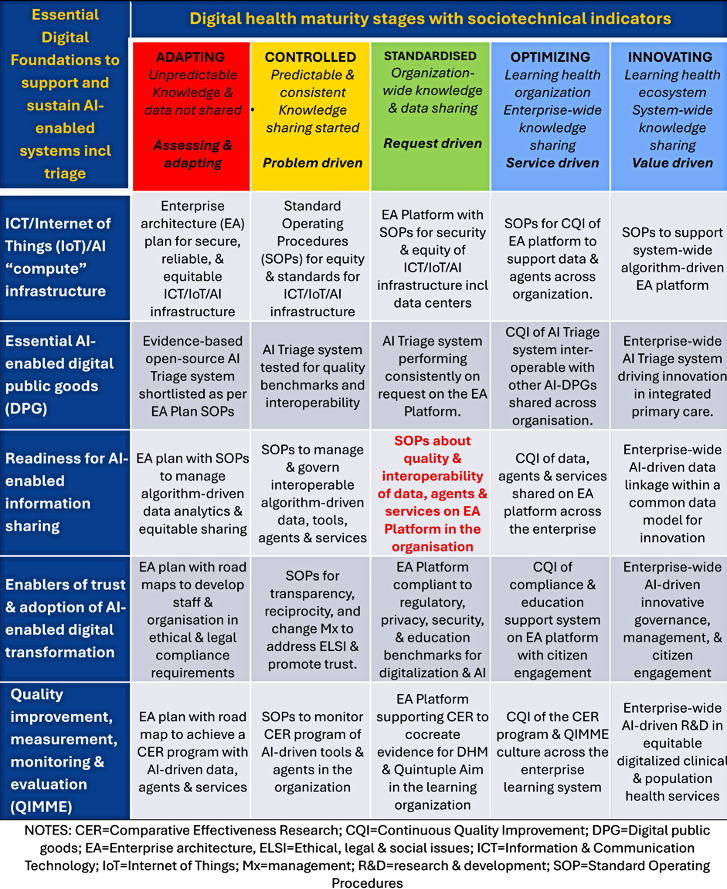
Indicators to assess readiness to support artificial intelligence (AI)–enabled triage and systems. incl: including; Mx: management; R&D: research and development; re: regarding.

Many organizations often skip maturity stages, usually because politicians favor “shiny new toys” over upstream essential but unglamorous standards and quality work to achieve an enterprise architecture that supports interoperability. This lack of well-implemented standards, along with proprietary systems in a competitive business environment, explains why interoperability remains a longstanding challenge and digital health interventions frequently fail to realize their potential benefits. It is also why the DHPMAT encourages the cocreation and use of open-source digital public goods.

Cocreation, including co-design, coimplementation, comonitoring, co-evaluation, and coimprovement over the development life cycle, is encouraged as a sociotechnical process to ensure that individual digital competencies and organizational DHM are improved concurrently with the Quintuple Aim [1,2,4]. Cocreating safe, useful, and usable cost-effective AI-enabled systems requires mutually trusting relationships among consumers, technology vendors, and health professionals in organizations and ecosystems. A fair and just regulatory framework that covers intellectual property (IP) and social capital is essential to cocreating, managing, governing, and sustaining digital assets equitably in local organizations and communities.

The balance between proprietary (private good) and open-source (public good) business models for digital health should be managed, especially in less-resourced countries. Proprietary organizations such as OpenAI have scraped the internet for diverse personal and public information, without formal consent from or payment to IP owners, to develop the generative pretrained transformer and large language model that underpin ChatGPT. Ironically, ChatGPT is now proprietary, and OpenAI is accusing DeepSeek, an open-source AI technology, of IP theft. OpenAI also illustrates the need for caution about AI-driven organizations with questionable business and humanitarian ethics [8].

A problem with terminology standards is the commodification of SNOMED-CT (Systematized Nomenclature of Medicine—Clinical Terms) and pharmaceutical classifications/vocabularies to be sold as proprietary terminology servers, adding financial and access barriers to semantically interoperable health information systems. In contrast, the WHO *International Statistical Classification of Diseases, 11th Revision* (*ICD-11*) is an open-source digital terminology public good and an alternative to SNOMED-CT. This private versus public good tension is inherent in every sector of the global economy [9].

Cocreating AI technology as a digital public good in a networked multilateral world is attractive. However, the corruption of efforts to develop digital public goods during COVID-19 by profit-seeking “big tech” and “big pharma” led to expensive suboptimal “shiny new toys” as well as vaccine, antiviral, and food apartheid. This continues to exacerbate inequity, morbidity, and mortality among people on the wrong side of the race, age, gender, poverty, and cultural divides [10].

While capitalist economies have greater inequality than socialist ones, both must deal with inequity as unintended sociotechnical consequences of development. Will the capitalist approach with privately owned corporations and “trickle-down-economics” or the socialist approach with state-controlled enterprises be more successful in minimizing digital health inequity [10]?

Equity-stratified AI-enabled health systems, including triage, must simultaneously improve the Quintuple Aim and DHM, especially access to high-quality and interoperable data, tools, agents, and services. This requires comprehensive sociotechnical and intersectoral approaches to investment, cocreation, management, governance, and quality improvement of digital tools to benefit individuals, organizations, and global ecosystems.

## Abbreviations

AI: artificial intelligence
DHM: digital health maturity
DHPMAT: Digital Health Profile and Maturity Assessment Toolkit
*ICD-11*: *International Statistical Classification of Diseases, 11th Revision*
IP: intellectual property
SNOMED-CT: Systematized Nomenclature of Medicine—Clinical Terms
WHO: World Health Organization
